# Supplementary material for: Effects of Drug Physicochemical Properties on In-Situ Forming Implant Polymer Degradation and Drug Release Kinetics
Source: Pharmaceutics. 2022 Jun 1;14(6):1188. doi: 10.3390/pharmaceutics14061188 (PMC9228340; doi:10.3390/pharmaceutics14061188)
Supplement: Supplementary file 1 [file pharmaceutics-14-01188-s001.zip › pharmaceutics-1722294-supplementary.pdf]

pharmaceutics

Supplementary Information

Effects of Drug Physicochemical Properties on In-Situ Forming Implant Polymer Degradation and Drug Release Kinetics

Jordan B. Joiner [1,†], Alka Prasher [2,†], Isabella C. Young [1], Jessie Kim [3], Roopali Shrivastava [2], Panita Maturavong-sadit [1] and Soumya Rahima Benhabbour [1,2,*]

[1] Division of Pharmacoengineering and Molecular Pharmaceutics, Eshelman School of Pharmacy, University of North Carolina at Chapel Hill, Chapel Hill, NC 27599, USA; jbjoiner@unc.edu (J.B.J.); iyoung4@live.unc.edu (I.C.Y.); panita@med.unc.edu (P.M.)
[2] Joint Department of Biomedical Engineering, University of North Carolina and North Carolina State University, Chapel Hill, NC 27599, USA; alkaprasher@gmail.com (A.P.); roopalis@email.unc.edu (R.S.)
[3] Eshelman School of Pharmacy, University of North Carolina at Chapel Hill, Chapel Hill, NC 27599, USA; jessiek@alumni.unc.edu
* Correspondence: benhabs@email.unc.edu
† These authors contributed equally to this work.

Citation: Lastname, F.; Lastname, F.; Lastname, F. Title. *Pharmaceutics* **2022**, *14*, 1188. https://doi.org/10.3390/pharmaceutics14061188

Academic Editor: Firstname Lastname

Received: date
Accepted: date
Published: 1 June 2022

Publisher's Note: MDPI stays neutral with regard to jurisdictional claims in published maps and institutional affiliations.

Copyright: © 2022 by the authors. Licensee MDPI, Basel, Switzerland. This article is an open access article distributed under the terms and conditions of the Creative Commons Attribution (CC BY) license (https://creativecommons.org/licenses/by/4.0/).

Supplementary Table S1. Summary of various ISFI formulations investigated to study the effects of drug physicochemical properties on drug release kinetics and polymer degradation over 90 days incubation in PBS at 37ºC. Drug concentration in ISFI formulations (mg/g) and in each depot (mg) injected into PBS was determined by HPLC analysis.

Drug	LogP	pKa	[drug] (mg/g)	Drug/depot (mg)
DRV	1.8	11.4	49.52 ± 2.78	1.28 ± 0.13
DTG	2.2	8.2	51.90 ± 5.1	1.21 ± 0.08
RTV	3.9	2.8	49.26 ± 3.71	1.51 ± 0.09
EFV	4.6	10.2	53.73 ± 3.96	1.72 ± 0.16
RPV	4.8	5.6	51.35 ± 3.04	1.73 ± 0.14
ETV	5.5	3.7	50.98 ± 1.87	1.60 ± 0.01
GEM	-1.4	3.4	50.10 ± 2.36	1.46 ± 0.04
3TC	-0.49	4.3	50.48 ± 3.18	1.57 ± 0.09
5FU	-0.89	8.02	51.77 ± 3.29	1.72 ± 0.13
RAL	-0.39	6.3	51.81 ± 2.79	1.61 ± 0.20
ZDV	0.05	9.7	53.44 ± 2.24	1.77 ± 0.15
IDA	0.2	9.5	51.20 ± 1.41	1.78 ± 0.08

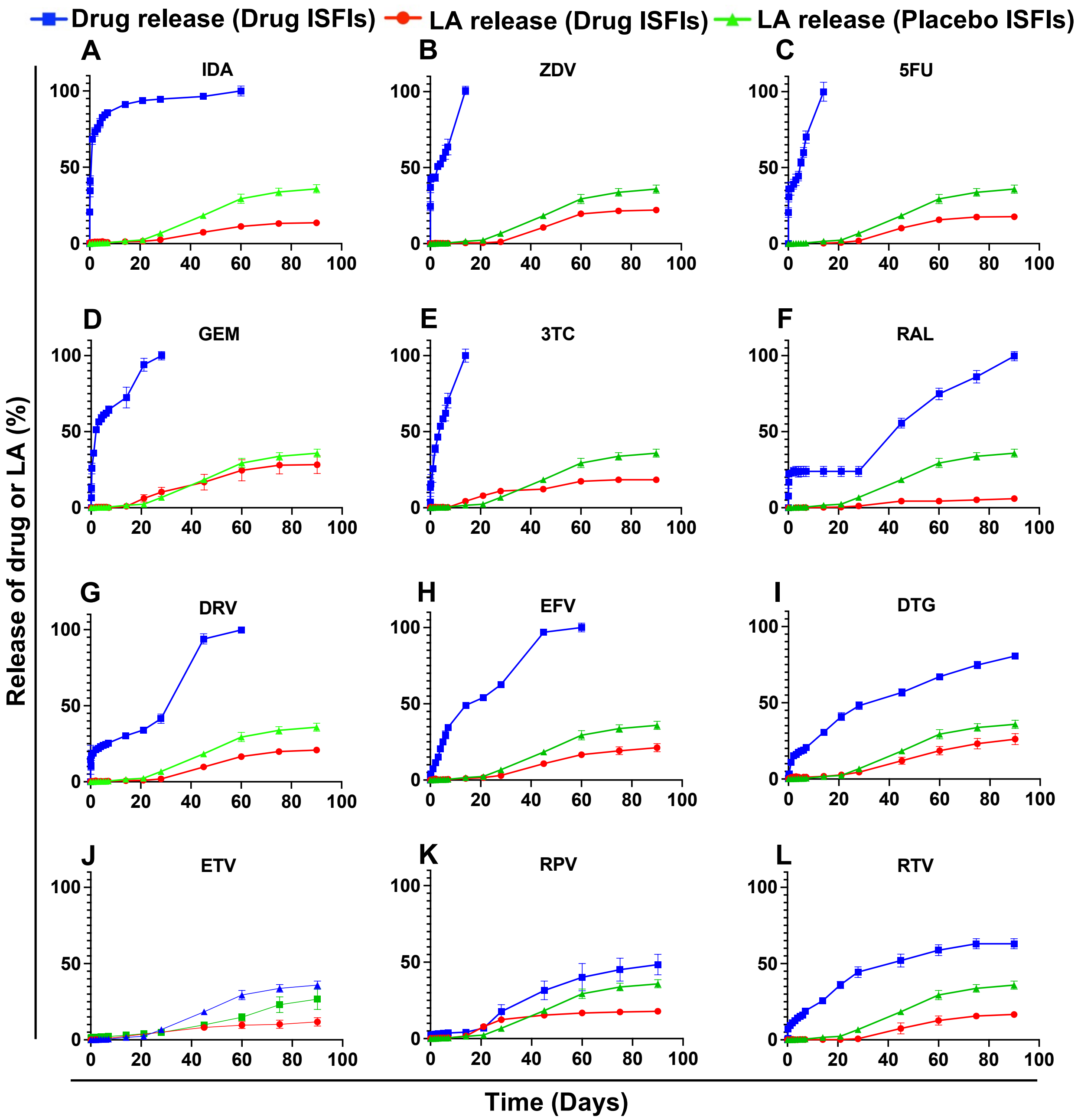

Supplementary Figure S1: In vitro drug release kinetics and PLGA degradation quantified by HPLC and lactic acid assay respectively over 90 days. % Drug release (blue), lactic acid release from drug-loaded ISFIs (red), and lactic acid release from placebo ISFIs (green). A) IDA ISFI (50 mg/g); B) ZDV ISFI (50 mg/g); C) 5FU ISFI (50 mg/g); D) GEM ISFI (50 mg/g); E) 3TC ISFI (50 mg/g); F) RAL ISFI (50 mg/g); G) DRV ISFI (50 mg/g); H) EFV ISFI (50 mg/g); I) DTG ISFI (50 mg/g); J) ETV ISFI 50 (mg/g); K) RPV ISFI (50 mg/g); L) RTV ISFI (50 mg/g).

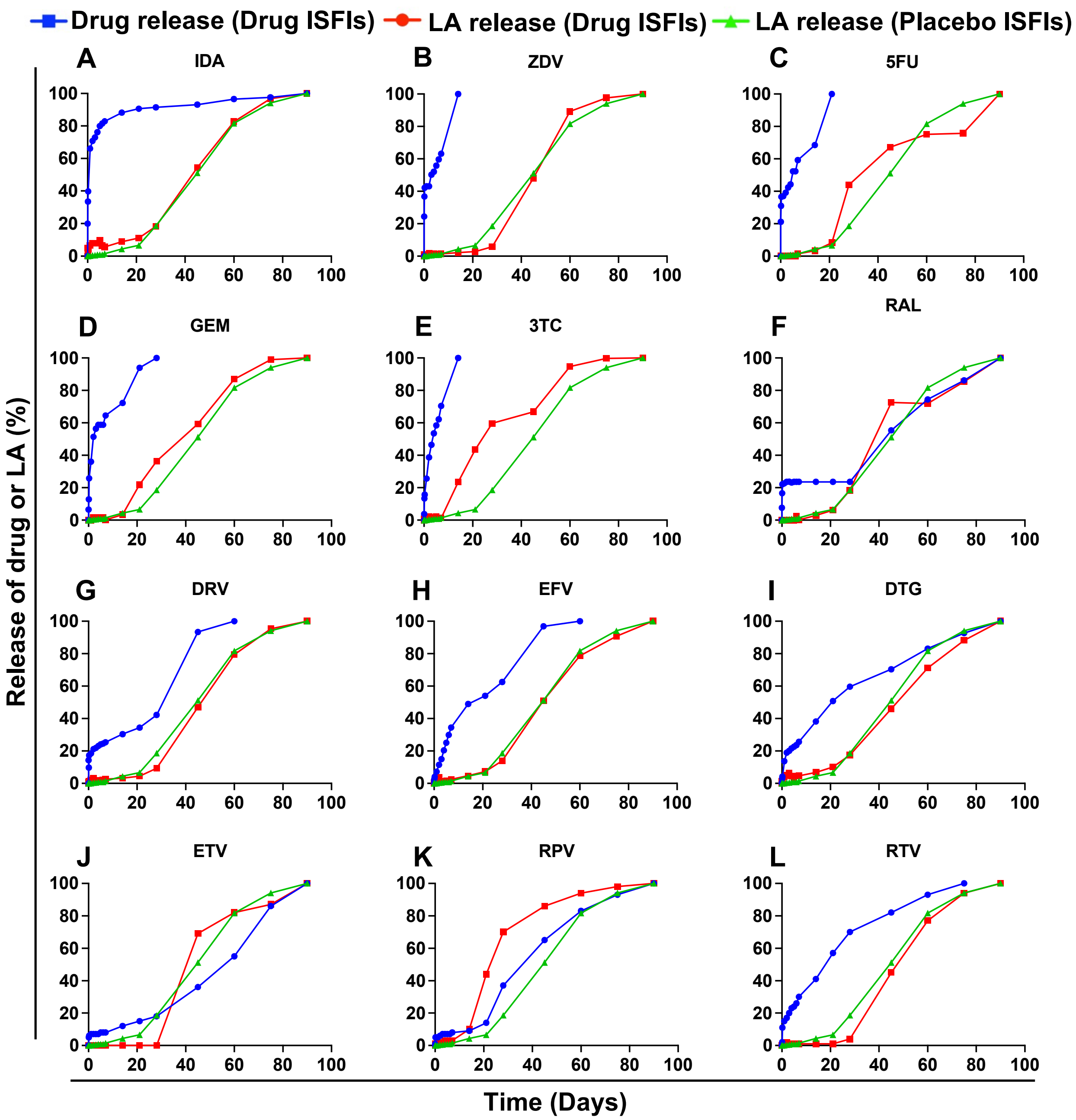

Supplementary Figure S2: In vitro drug release kinetics and PLGA degradation quantified by HPLC and lactic acid assay respectively over 90 days. Drug release normalized to % release at day 90 (blue), lactic acid release from drug-loaded ISFIs normalized to % release at day 90 (red), and lactic acid release from placebo ISFI normalized to % release at day 90 (green). A) IDA ISFI (50 mg/g); B) ZDV ISFI (50 mg/g); C) 5FU ISFI (50 mg/g); D) GEM ISFI (50 mg/g); E) 3TC ISFI (50 mg/g); F) RAL ISFI (50 mg/g); G) DRV ISFI (50 mg/g); H) EFV ISFI (50 mg/g); I) DTG ISFI (50 mg/g); J) ETV ISFI 50 (mg/g); K) RPV ISFI (50 mg/g); L) RTV ISFI (50 mg/g).

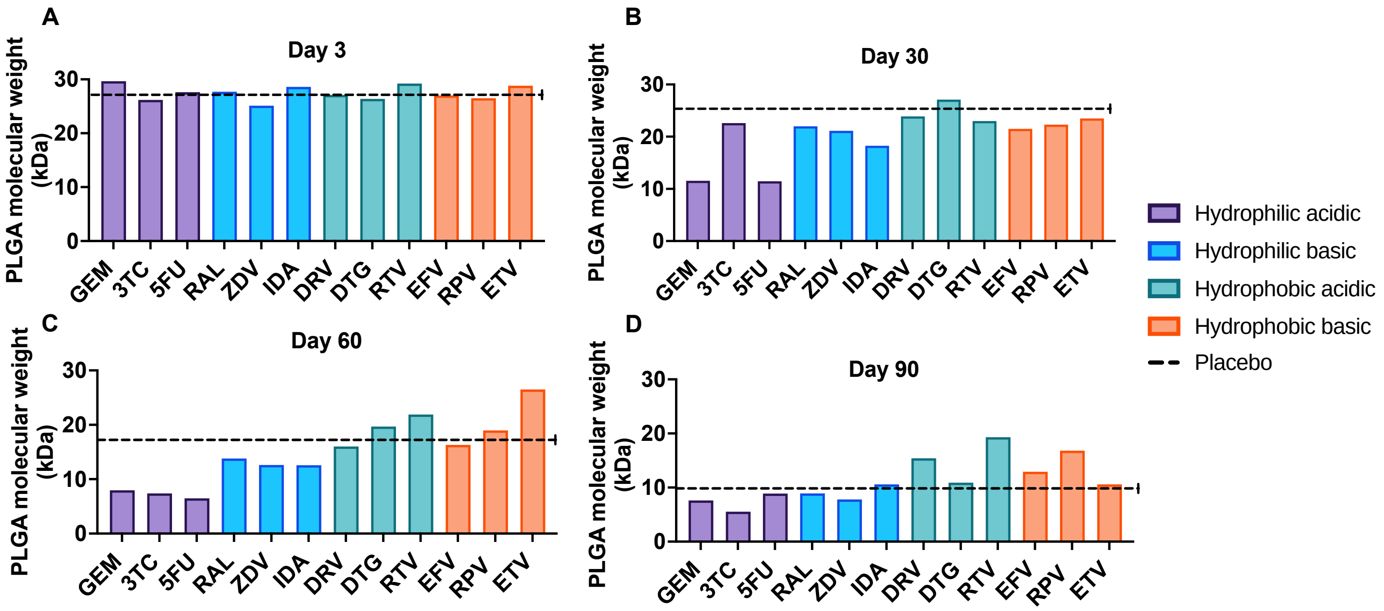

Supplementary Figure S3: Molecular weight of PLGA (kDa) as measured by GPC analysis for each drug at (A) day 3 (B) day 30 (C) day 60 (D) day 90.
